# Use of a Selected *Leuconostoc Citreum* Strain as a Starter for Making a “Yeast-Free” Bread

**DOI:** 10.3390/foods8020070

**Published:** 2019-02-13

**Authors:** Palmira De Bellis, Carlo Giuseppe Rizzello, Angelo Sisto, Francesca Valerio, Stella Lisa Lonigro, Amalia Conte, Valeria Lorusso, Paola Lavermicocca

**Affiliations:** 1 Institute of Sciences of Food Production (ISPA), National Research Council (CNR), Via G. Amendola 122/O, 70126 Bari, Italy; angelo.sisto@ispa.cnr.it (A.S.); francesca.valerio@ispa.cnr.it (F.V.); lisa.lonigro@ispa.cnr.it (S.L.L.); valeria.lorusso19@gmail.com (V.L.); paola.lavermicocca@ispa.cnr.it (P.L.); 2 Department of Soil, Plant and Food Science, University of Bari Aldo Moro, Via G. Amendola 165/A, 70126 Bari, Italy; carlogiuseppe.rizzello@uniba.it; 3 Department of Agricultural Sciences, Food and Environment, University of Foggia, Via Napoli 25, 71122 Foggia, Italy; amalia.conte@unifg.it

**Keywords:** “yeast-free” bread, liquid sourdough, selected starter, *Leuconostoc citreum*, *Weissella confusa*

## Abstract

The aim of this study was the characterization and selection of bacterial strains suitable for the production of a “yeast-free” bread. The strains *Leuconostoc citreum* C2.27 and *Weissella confusa* C5.7 were selected for their leavening and acidification capabilities and individually used as starters in bread-making tests. Liquid type-II sourdoughs, singly inoculated with the two selected strains, were characterized and employed for bread-making, through the set-up of a biotechnological protocol without the use of baker’s yeast as a leavening agent. Aiming to verify the ability of the selected strains to dominate the fermentation process, bacteria and yeasts were isolated from liquid sourdoughs and doughs, genetically characterized and identified. Both the selected strains were suitable for the production of bread, even if *L. citreum* C2.27 showed the highest leavening capacity and was able to dominate the dough microbiota. The effects of different salt concentrations on the selected strain performances were also investigated. The applicability of the developed protocol, adapted for the production of the typical Apulian bread, “puccia”, and the suitability of the strain *L. citreum* C2.27 were confirmed at pilot scale in an industrial bakery. The puccia bread, which was produced with the liquid sourdough fermented with *L. citreum* C2.27, without baker’s yeast and salt, was similar in appearance to the conventional product containing baker’s yeast and was judged positively by a sensory analysis.

## 1. Introduction

The bakery sector is one of the most important sectors in the food industry, as bread and other baked goods are fundamental foods of the human diet and are consumed daily by most people. Although simple in their composition, bakery products are subject to a rising demand for innovation by their producers, which also depend on the consumers’ demand for foods with healthier nutritional properties. In particular, there is an increasing trend in the market towards foods without ingredients that are considered as a source of adverse reactions. Among them, the request for yeast-free bakery products is included, as baker’s yeast has been related, also in recent studies [1,2], to hypersensitivity reactions [3]. In fact, some components of the cell wall in baker’s yeast (*Saccharomyces cerevisiae*) have already been recognized as antigens in patients with chronic inflammatory bowel diseases (IBDs), which include ulcerative colitis (UC) and Crohn’s disease (CD). In particular, different immune responses to the phosphopeptidomannans of the *S. cerevisiae* cell wall were observed between UC and CD. Therefore, anti-*S. cerevisiae* antibodies (ASCA) are considered a suitable marker for aiding the differentiation of those syndromes and the diagnosis of CD [4]. Moreover, patients with CD reported adverse reactions more frequently associated to bakery products containing baker’s yeast than to products obtained with sourdough or without baker’s yeast [5]. Nevertheless, ASCA antibodies have been also associated with other autoimmune diseases [2,6] and obesity [7]. 

Recently, Musatti et al. [8] studied alternatives to leavening with baker’s yeast. They explored an unconventional bacterial association between *Zymomonas mobilis*, isolated from fermented agave sap, and *Lactobacillus sanfranciscensis* for dough leavening. Despite the efficacy of that microbial association, the study was performed only in a model dough and did not include an evaluation of bread-making experiments. Overall, numerous advantages of performing the starter selection among microorganisms isolated from the same environment in which they will be applied have been reported [9]. Indeed, many strains selected in vitro for their valuable characteristics are often not well adapted to the cereal environment and consequently do not have the capacity to compete with the endogenous microbiota and have difficulty in dominating the sourdough ecosystem [10]. 

As in the case of other fermented products (meat, enological and dairy products), the interest in the use of commercial starters is also increasing in the bakery sector. Starter strains should standardize the production process, improving at the same time the features of the bakery products. Nevertheless, few bakery applications of starter lactic acid bacteria (LAB) at an artisanal and industrial level are known, while there are several studies reporting the selection of LAB strains for peculiar technological, nutritional or sensory implications, as well for related laboratory bread-making trials [11,12,13,14,15]. It should also be taken into account that in many applications selected LAB were used for the production of sourdoughs, which contain a high final population of yeasts at the end of the backslopping procedure [16].

In recent years, besides the need for proper starters, industrial bakeries also need to produce leavened products with less laborious, faster and more controllable processes than those based on the use of traditional sourdough. In this perspective, liquid sourdough offers several advantages: Greater flexibility of use, easier control of fermentation, easier procedures to manage and reproduce, the possibility of using it in different types of baked goods and the possibility of rapid preparation, also using selected and frozen or freeze-dried preserved microbial cultures [17].

In this context, this study aimed to develop a biotechnological protocol for the production of “yeast-free” bread by using a ready-to-use liquid sourdough. A selection of suitable LAB strains, (mainly based on their leavening ability) was carried out, and by using them as starters, yeast-free doughs and breads were produced and characterized. The biotechnological protocol was then applied at pilot plant scale for the production of a “yeast-free” variant of the Apulian typical bread, “puccia salentina”.

## 2. Materials and Methods 

### 2.1. Bacterial Strains and Growth Conditions 

Twelve LAB strains were used in this study (Table 1). The choice of these strains was based on their characteristics and origin. The strains belonged to eight different species and were homofermentative or heterofermentative. Six strains, isolated from Italian durum wheat semolina or sourdough, belonged to the Culture Collection of the Institute of Sciences of Food Production of National Research Council (ISPA-CNR). Four strains, isolated from sourdough, belonged to the Culture Collection of Department of Soil, Plant and Food Science of Bari University (DiSSPA). Two type strains were from DSMZ microbial collection (Leibniz Institute DSMZ-German Collection of Microorganisms and Cell Cultures, Braunschweig, Germany). The strains were routinely propagated in a de Man–Rogosa–Sharpe (MRS) broth (Oxoid Ltd, Basingstoke, UK), except those of the species *Lactococcus lactis*, *Weissella cibaria* and *Lactobacillus rossiae*, which were cultivated in a modified MRS (mMRS) broth (obtained by adding 1% (*w*/*v*) maltose and 5% (*v*/*v*) fresh yeast extract to MRS, pH 5.6) [18]. The strains were 2% (*v*/*v*) inoculated in the mMRS/MRS broth and subcultured twice at 30 °C for 24 h in the same medium before each experiment. For long-term storage, 1 mL aliquots of fresh cultures with 20% Bacto glycerol (Difco) were frozen at −80 °C. 

### 2.2. Selection of Lactic Acid Bacteria 

In order to characterize and select LAB strains suitable as starters for the preparation of dough without baker’s yeast, 12 LAB strains were singly inoculated in doughs and characterized. In particular, each strain was cultivated for 24 h, then cells were harvested by centrifugation (9000 g for 10 min at 4 °C), washed twice with a sterile saline solution (NaCl 0.85%, *w*/*v*) and re-suspended in sterile distilled water (SDW) at a final OD600 of 2.3, corresponding to a cell density of ca. 9 log cfu/mL. Cell density of each strain was confirmed by plate counts. Three hundred grams of dough (dough yield (DY), (dough weight × 100/flour weight) = 160) was prepared by mixing for 5 min wheat flour (62.5% *w*/*w*), sterile distilled water (32.5% *v*/*w*) and the bacterial suspension (5% *v*/*w*) (final cell density in the dough ca. 7 log cfu/g of dough). The dough was divided into aliquots and incubated at 30 °C. After 8, 16 and 24 h of fermentation, the volume increase (ΔV, mL), the pH drop (ΔpH), and the total titratable acidity (TTA) were measured [20]. The experiment was repeated three times, and three independent aliquots for each batch were analyzed.

### 2.3. Molecular Characterization of LAB Strains

The molecular characterization of 12 LAB strains was carried out to allow their monitoring during the bread-making process. Bacterial DNA was extracted from overnight cultures grown in the mMRS/MRS broth (Oxoid, UK) at 30 °C, using a Clonsaver Card Kit (Whatman, Maidstone, UK) and analyzed by repetitive extragenic palindromic PCR (REP-PCR) [21]. In order to check the strain-specificity of the obtained REP-PCR profiles, they were compared with each other and with those of 30 other LAB strains from the ISPA collection.

### 2.4. Preparation of Liquid Sourdoughs and Bread-Making at Laboratory Scale

Based on their acidification and leavening capabilities (Table 2), two very promising strains, namely *L. citreum* C2.27 and *W. confusa* C5.7, were selected and singly used in the laboratory for bread-making tests. The bacterial suspensions in SDW were prepared as described in Section 2.2.

Liquid sourdoughs, singly inoculated with *L. citreum* C2.27 (S_C2.27_) or *W. confusa* C5.7 (S_C5.7_), were prepared by mixing 50 g of wheat flour (17% *w*/*w*), 175 mL of sterile tap water (58% *v*/*w*) and 75 mL of bacterial suspension (25% *v*/*w*), corresponding to a cell density of ca. 8 log cfu/mL, thus having a DY of 600. Then, the mixture was incubated at 30 °C under stirring conditions (110 rpm) for 16 h. 

Bread doughs D_C2.27_ or D_C5.7_, with a DY of ca. 160, were produced by mixing wheat flour (450 g, 60% *w*/*w*), sterile tap water (75 mL, 10% *v*/*w*) and S_C2.27_ or S_C5.7_ (225 mL, 30% *v*/*w*). Bread doughs with 2% (*w*/*w*) baker’s yeast, corresponding to a final yeast density of ca. 8 log cfu/g, and without liquid sourdough, were used as controls (D_CTR_). Aliquots (200 g) of each dough were placed in rectangular steel pans, fermented at 30 °C for 6 h (D_C2.27_, D_C5.7_) or 1.5 h (D_CTR_) and baked in an electric oven (Ardes, Milano, Italia) at 190 °C for 20 min. Three independent bread-making tests were carried out. 

### 2.5. Biochemical Characterization of Laboratory Sourdoughs and Bread Doughs

After fermentation, the ΔpH and TTA were determined for the liquid sourdoughs (S_C2.27_ and S_C5.7_) and bread doughs (D_C2.27_, D_C5.7_ and D_CTR_). For the bread doughs, the volume increase (ΔV, mL) was also measured [20]. 

To quantify organic acids in the sourdough and bread dough, samples were prepared and analyzed by High Performance Liquid Chromatography (HPLC), as reported by Valerio et al. [22]. Quantification of the organic acids was performed by integrating calibration curves obtained from the relevant standards. The limit of detection (LOD) and the limit of quantification (LOQ) were calculated considering a signal-to-noise ratio (S/N) of 3 and 6. LOD values were 0.421 mmoli/kg and 0.127 mmol/kg for lactic acid in sourdough and bread dough, respectively. For acetic acid, the LOD values were 0.138 mmoli/kg and 0.233 mmol/kg in sourdough and bread dough, respectively. The LOQ values corresponded to 2 × LOD. The quotient of fermentation (QF) was determined as the molar ratio between the lactic and acetic acids. 

The concentration of peptides and free amino acids (FAAs) was determined in the water/salt-soluble extracts (WSEs) of doughs to evaluate the degree of proteolysis in the native proteins of doughs. The WSEs of doughs were prepared according to Weiss et al. [23]. For the peptide analysis, WSEs were treated with trifluoroacetic acid (0.05% *w*/*v*) and subjected to dialysis (cut-off 500 Da) to remove proteins and FAA, respectively. Then, the concentration of peptides was determined by the o-phtaldialdehyde (OPA) method, as described by Church et al. [24]. The FAAs were analyzed by a Biochrom30 series Amino Acid Analyzer (Biochrom Ltd, Cambridge Science Park, Cambridge, UK) with a Na-cation exchange column (20 × 0.46 cm internal diameter), as reported in Rizzello et al. [25].

### 2.6. Microbiological Analyses and Monitoring of Selected Strains

The liquid sourdoughs were directly subjected to decimal dilutions and plating, while 20 g of bread doughs were previously added to a 180 mL sterile NaCl solution (0.85%) and homogenized in a Stomacher (Seward, London, United Kingdom) for 2 min. The resulting suspensions were plated on a mMRS agar (Oxoid, Basingstoke, Hampshire, UK), supplemented with 100 mg/L of cycloheximide (Merck, Darmstadt, Germany) and incubated at 30 °C for 48 h to determine the LAB counts. The resulting suspensions were also plated on a Sabouraud dextrose agar (Oxoid, Basingstoke, Hampshire, UK), supplemented with 200 mg/L chloramphenicol (Sigma, Milan, Italy) and incubated for 72 h at 25 °C for yeast and mould counts.

The monitoring of the starters (*L. citreum* C2.27 or *W. confusa* C5.7) in liquid sourdoughs and bread doughs was carried out on the basis of their strain-specific REP-PCR profiles by analyzing 20% of the colonies from the countable mMRS agar plates, as previously described [21]. 

### 2.7. Identification of LAB and Yeasts

The liquid sourdoughs and bread doughs indicated in Section 2.6 were characterized by monitoring the starter strains and identifying the other presumptive LAB strains from countable mMRS agar plates. Therefore, bacterial isolates representative of each REP-PCR profile, different from the patterns of the starter strains, were identified by the sequencing of the almost complete 16S rRNA gene, as previously described in [21], using an ABI Prism 3730xl DNA Analyzer (Thermo Fisher Scientific, Waltham, MA, USA). The species *Lb. plantarum*/*Lb. paraplantarum* and *Lb. paracasei* were also identified by multiplex-PCR methods, as described by Torriani et al. [26] and Ventura et al. [27], respectively. Moreover, in order to characterize and identify the yeasts present in the same liquid sourdoughs and bread doughs, 20% of the colonies present on the countable Sabouraud dextrose agar plates (Oxoid, UK) were purified. The DNA was extracted from 1.5 mL cultures grown in YEPG (yeast extract 1% *w*/*v*, peptone 1% *w*/*v* and dextrose 2% *w*/*v*) at 25 °C for 24 h, using the Wizard Genomic DNA Purification kit (Promega Corporation, USA), after which it was amplified by the oligonucleotide (GTG)^5^ [28]. The PCR reactions were performed as previously described [29]. Representative isolates of each PCR profile were identified through the amplification and sequencing of the D1/D2 domain of the 26S rDNA, using the primers NL1 and NL4 [30]. 

### 2.8. Characterization of the Laboratory Breads 

The analyses of pH and TTA were carried out as previously reported [20]. The lactic and acetic acids were measured as indicated by Valerio et al. [22]. Water activity (a_w_) was measured with AcquaLab (Decagon Devices, Inc., Pullman, WA, USA). The specific volume of the breads was measured by the BVM test system (TexVol Instruments, Viken, Sweden). Instrumental textural profile analysis (TPA) was carried out with a TVT-300XP Texture Analyzer (TexVol Instruments), equipped with the cylinder probe P-Cy25S. For the analysis, the crust of the baked loaves was not removed. The settings were selected as follows: Test speed 1 mm/s, 30% deformation of the sample and one compression cycle [14]. The data were processed with the Texture Analyzer TVT-XP 3.8.0.5 software (TexVol Instruments, West Sussex, UK), obtaining the following textural parameters: Hardness (maximum peak force), fracturability (the first significant peak force during the probe compression of the bread), and resilience (ratio of the first decompression area to the first compression area). 

The crumb grain of the breads was evaluated after 24 h of storage using image analysis technology. Images of the sliced breads were captured using an image scanner (Amersham Pharmacia Biotech, Uppsala, Sweden). The images were scanned full-scale at 300 dots per inch and analyzed in grayscale (0–255). Image analysis was performed using the UTHSCSA ImageTool program (Version 2.0, University of Texas Health Science Centre, San Antonio, Texas, USA, available by anonymous FTP from maxrad6.uthscsa.edu). A threshold method was used for differentiating gas cells and non-cells [31]. Analysis was carried out on two sub-images of 500 × 500 pixels (field of view) selected from within the bread slice. Two slices were analyzed per treatment.

The chromaticity coordinates of the samples (obtained by a Minolta CR-10 camera) were reported in the form of a color difference, dE*_ab_, as follows:

(1) $$dE_{ab}^{*}=\sqrt{{\left(dL\right)}^{2}+{\left(da\right)}^{2}+{\left(db\right)}^{2}}$$

where *dL*, *da* and *db* are the differences for L, a and b values between the sample and reference (a white ceramic plate, where L = 94.8, a = 0.4 and b = 4.16).

### 2.9. Effect of Salt on Starter Performances

Aiming to investigate the effect of the salt concentration on selected starter performances, different percentages of salt were tested. In particular, doughs inoculated with *L. citreum* C2.27, containing 2% (the percentage usually used in bakeries), 1.5%, 1%, 0.5% and 0% (*w*/*w*) salt (NaCl) were prepared as described above (Section 2.2) and monitored at 1 h intervals for ΔV and ΔpH during 8 h of incubation at 30 °C.

### 2.10. Bread-Making at a Pilot Plant Scale

The strain *L. citreum* C2.27 was selected for bread-making tests at pilot plant scale, mainly based on the microbiological characteristics of laboratory liquid sourdoughs and bread doughs, and the technological features of the breads. The biotechnological protocol developed in the laboratory was also evaluated at a pilot plant level in the industrial bakery Valle Fiorita (Ostuni, Italy), and adapted for the production of “puccia salentina”, a typical Apulian bread. *L. citreum* C2.27 was provided to the bakery as a pellet obtained by the centrifugation of a bacterial suspension in sterile NaCl solution (0.85%) at 9000 rpm for 10 min. In the bakery, the liquid sourdough was prepared in a 100 l FN120 automatic bioreactor (Novasilos, Forlì, IT) by mixing soft wheat flour type “00” (17% *w*/*w*) with tap water (83% *v*/*w*). The LAB cells were re-suspended in water before mixing. The inoculum was ca. 8 log cfu/mL, and fermentation was carried out at 30 °C for 16 h under continuous stirring.

The puccia bread doughs (DY ca. 160) were prepared by mixing durum wheat semolina (ca. 30% *w*/*w*), wheat flour (ca. 30% *w*/*w*), tap water (ca. 6% *v*/*w*), olive oil (1.5% *v*/*w*) and liquid sourdough S_C2.27_ (30% *v*/*w*). Puccia bread doughs obtained using sourdough were prepared with (1.5% *w*/*w*; D1.5_C2.27_) or without salt (D0_C2.27_). Puccia bread doughs, not containing liquid sourdough, were also prepared as controls using baker’s yeast, (2% *w*/*w*) with (1.5% *w*/*w*, D1.5_CTR_) or without salt (D0_CTR_). Dough portions of 130–135 g were placed in plastic trays for the leavening process. In particular, incubation was carried out at 30 °C for 4 h (1.5 h for the controls) at 75% relative humidity. The doughs were baked in a tunnel oven coated by refractory stone at 350 °C for 90 s. The sourdoughs and puccia doughs were characterized as described above.

### 2.11. Sensory Analysis of Puccia Bread 

Sensory analysis of puccia bread samples produced at a pilot plant level in the industrial bakery Valle Fiorita (see above) was performed, as previously described by Valerio et al. [22], with some modification, by a trained panel group composed of sixteen assessors (8 male and 8 female, mean age: 35 years, range: 18–54 years). 

The sensory attributes, scored with a scale from 1 to 9, were: Color (from bright yellow = 1 to dark brown = 9), hardness (resistance to first finger compression, from very low = 1 to high = 9), elasticity (ability of the sample to regain its original form after finger pressure, from low elasticity = 1 to very elastic = 9), pore size (from small = 1 to large = 9), pore homogeneity (presence of pores of the same size, from not homogeneous = 1 to very homogeneous = 9), aroma (degree of perceived aroma, assessed by smelling the sample, from low intensity = 1 to high intensity = 9), taste (degree of perceived taste, assessed by chewing the sample, from low intensity = 1 to high intensity = 9) and aftertaste (degree of perceived intensity of aftertaste after chewing the sample, from low intensity = 1 to high intensity = 9). The sensory attributes were discussed with the assessors during two introductory training sessions (1 session/day, 1 h/session). The samples were served in a random order and evaluated in two replicates by all panelists. Before the sensory evaluation, the loaves were thawed at room temperature for 5 h, then sliced using an electric slicing knife (thickness of 15 mm, Atlantic, Calenzano, Firenze, Italy), without removing the crust. The slices were cut into 4 pieces and each panelist received 2 pieces per sample. 

### 2.12. Statistical Analysis

The data are presented as mean values ± standard error. Statistical analysis of the data was performed using STATISTICA (data analysis software system), version 10 (StatSoft, Inc., Tulsa, OK, USA). The data concerning volume, pH, TTA, organic acids, free amino acids, textural profile analysis, image analysis, color analysis, microbial counts and sensory analysis were compared by applying a one-way ANOVA followed by Tukey’s test to determine significantly different values (*p* < 0.05).

## 3. Results

### 3.1. Selection of the Starters

Aiming to select LAB strains to be used as starters for dough fermentation, the leavening and acidification capabilities of 12 strains (Table 1) were evaluated and monitored during 24 h of incubation (Table 2).

Considering the aim of the study, the volume increase (ΔV, mL) of the doughs during fermentation was the most important parameter taken into account. The doughs inoculated with the different strains showed a different leavening capacity during fermentation. Some strains showed good leavening capacity after 8 hours, while others did after many hours, and others did not determine a significant change of the dough volume during fermentation time.

Among the tested LAB, *L. citreum* C2.27 showed a remarkable leavening capacity after 8 h and a good acidifying capacity during fermentation. Other strains with good dough leavening capacity after 8 h, besides giving a good acidity to the doughs, were *W. confusa* C5.7 and DSM20196, and *W. cibaria* C21.4. No significant (*p* > 0.05) differences in ΔV values were found between the doughs inoculated with *W. confusa* C5.7 and *W. cibaria* C21.4, however, *W. confusa* C5.7 determined a greater ΔV over time and also a good acidification. On the basis of these results, and considering as positive the speed in leavening and acidification, *L. citreum* C2.27 and *W. confusa* C5.7 were selected and used for the following tests.

### 3.2. Genotypic Characterization of LAB Strains

**Figure 1:**
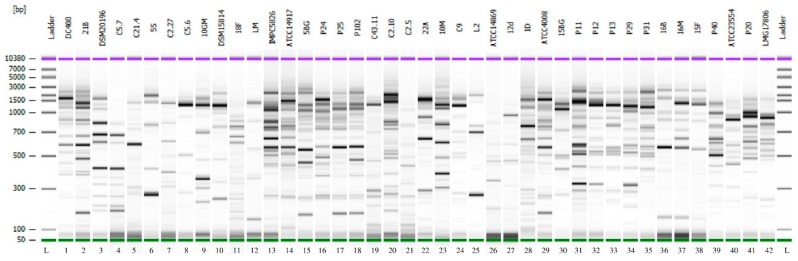
REP-PCR fingerprints of the 12 LAB strains in this study compared with other strains of the ISPA-CNR collection. The patterns were obtained using primers REP-1R-Dt/REP-2R-Dt and shown as a gel-like image using the Agilent Expert software. 1–12, REP-PCR profiles of the strains evaluated in this study for the production of doughs without baker’s yeast (Table 1). 13–42, REP-PCR profiles of strains belonging to different species: 13–18, *Lb. plantarum*; 19–21, *W. cibaria*; 22 and 23, *L. citreum*; 24, *Lc. Lactis*; 25, *Lb. rossiae*; 26–28, *Lb. brevis*; 29–35, *Lb. pentosus*; 36–38, *Lb. alimentarius*; 39 and 40, *Lb. paracasei*; 41 and 42, *Lb. casei*. L: DNA 7500 ladder.

REP-PCR was used in order to obtain an electrophoretic pattern suitable for the identification of the tested strains in the liquid sourdough and bread dough samples. The results of the analysis are shown in Figure 1. The method differentiated all the strains considered, which also showed profiles different from those of the other LAB of the ISPA-CNR collection. The obtained molecular fingerprints allowed for the distinguishment of strains belonging to different species, but also strains of the same species. 

### 3.3. Bread-Making at Laboratory Scale

#### 3.3.1. Characterization of Liquid Sourdoughs and Bread Doughs

A two-step protocol for the production of bread without baker’s yeast addition was developed. It included two incubation steps, the first of which aimed to prepare a liquid sourdough inoculated with the selected starter (step I) and the second’s aim was the preparation of the dough to be used for bread making (step II). *L. citreum* C2.27 and *W. confusa* C5.7 were singly used as a starter. The results of the characterization of sourdoughs and bread doughs are reported in Table 3.

Both the strains caused a relevant acidification of the sourdoughs. Lactic and acetic acids were at higher concentrations in S_C2.27_ than in S_C5.7_ (*p* < 0.05), even if their quotients of fermentation were not significantly different (*p* > 0.05).

The use of the liquid sourdoughs caused ΔpH and TTA values higher for D_C2.27_ and D_C5.7_ compared to the control. Lactic acid concentration was higher in D_C2.27_ than in D_C5.7_ (*p* < 0.05), while acetic acid was detected only in D_C2.27_ (QF of ca. 2). Both the organic acids were not detected (<LOD) in the control dough.

*L. citreum* C2.27 and *W. confusa* C5.7 caused the increase in volume in the bread dough. In particular, ΔV of D_C2.27_ was almost 2-fold higher than that observed for D_C5.7_. As expected, the dough started with baker’s yeast showed the highest ΔV (*p* < 0.05).

**Figure 2:**
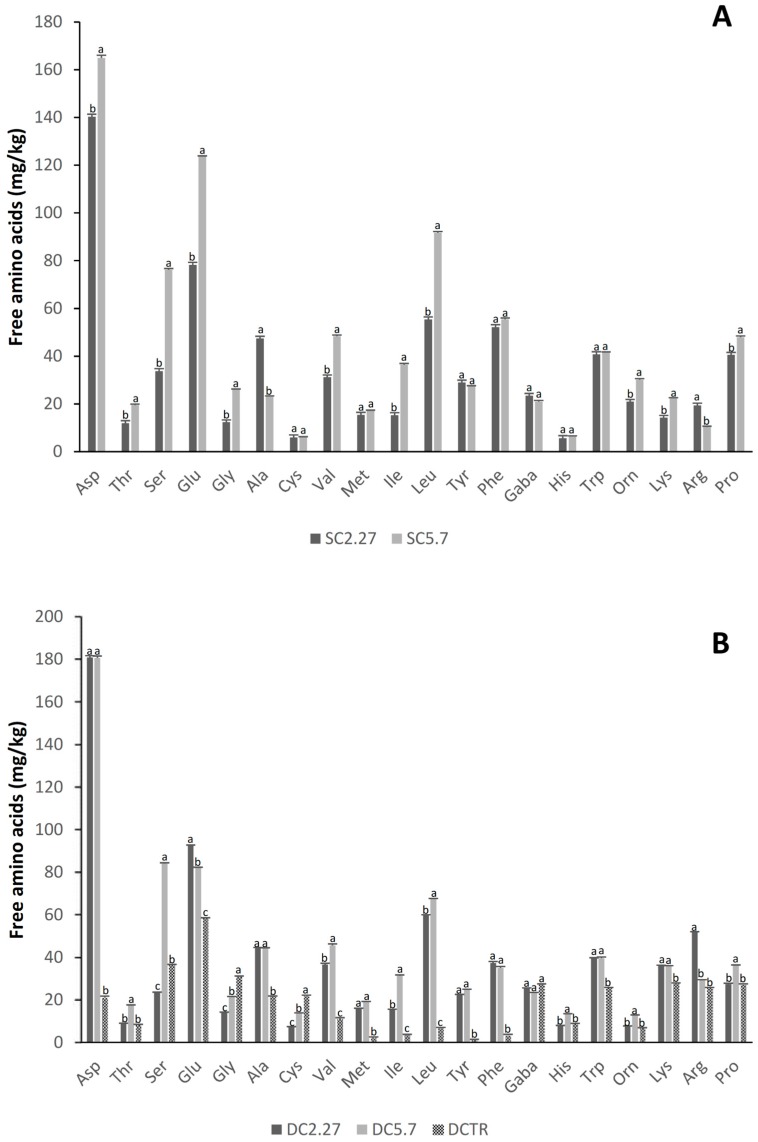
Concentration of free amino acids and their derivatives (mg/kg) in liquid sourdoughs (S_C2.27_ and S_C5.7_) inoculated with starter strains (**A**) and bread doughs obtained with liquid sourdough (D_C2.27_ and D_C5.7_) or with baker’s yeast (D_CTR_) (**B**) in laboratory tests. Data are the means of three independent analyses. The three-letter amino acid code (IUPAC) is used. ^a–c^ Values with different superscript letters within the same amino acid differ significantly (*p* < 0.05). Bars of standard deviation are also represented.

Peptide concentration in S_C5.7_ was slightly but significantly (*p* < 0.05) higher than in S_C2.27_. In addition, the total FAAs were significantly (*p* < 0.05) higher in S_C5.7_ compared to S_C2.27_ (Table 3). In particular, Asp, Glu and Leu were the amino acids found at the highest concentrations in both the sourdoughs (Figure 2A). Several amino acids, such as Asp, Thr, Ser, Glu, Gly, Val, Ile and Pro were at significantly (*p* < 0.05) higher concentrations in S_C5.7_ than in S_C2.27_, in particular, the concentrations of Ser, Gly and Ile were more than double in S_C5.7_ compared to S_C2.27_. On the contrary, Ala and Arg were found at significantly (*p* < 0.05) higher levels in S_C2.27_ than in S_C5.7_ (Figure 2A). 

When sourdoughs were used for bread-making, the concentrations of peptides and total FAAs of bread doughs significantly (*p* < 0.05) increased compared to the baker’s yeast control, D_CTR_ (Table 3). Peptides in D_C2.27_ and D_C5.7_ were 2 times higher than in the control. Accordingly to the concentrations found in the sourdoughs, the total amount of FAAs in D_C2.27_ and D_C5.7_ were significantly (*p* < 0.05) higher than in D_CTR_. Figure 2B reports the individual concentrations of FAAs. Similar concentrations of Thr, GABA, His, Orn and Pro were found in D_C2.27_ and D_CTR_, while with the exception of Gly, Cys, GABA and Arg, all the FAAs were found at significantly (*p* < 0.05) higher levels in D_C5.7_ than in D_CTR_ (Figure 2B). Lys, the major limiting amino acid in wheat flour, was ca. 30% higher in the sourdough breads compared to the control.

#### 3.3.2. Microbiological and Molecular Characterization of LAB and Yeasts

Liquid sourdoughs (S_C5.7_, S_C2.27_) and bread doughs (D_C5.7_, D_C2.27_) showed cell densities of presumptive LAB (10^8^ cfu/g) significantly higher than dough with baker’s yeast (10^5^ cfu/g) (Table 4). In S_C2.27_ and D_C2.27_, the REP-PCR analysis showed only the presence of *L. citreum* C2.27, corresponding to 100% of the analyzed population. On the contrary, a *Lb. plantarum* strain was found in S_C5.7_ and D_C5.7_, together with the added starter. Despite a significantly lower density of LAB in D_CTR_ compared to the other doughs, 9 LAB strains of different species were found.

Overall, yeasts were not detected in sourdoughs and moulds were in very low densities (Table 4). Moreover, yeasts and moulds were not detected in bread doughs inoculated with *L. citreum* C2.27, while a low density of a single strain of the yeast *Filobasidium magnum*/*floriforme* (100% identity) was detected in D_C5.7_. As expected, the control dough (D_CTR_) showed a very high count of yeasts identified as *S. cerevisiae* (100% identity).

#### 3.3.3. Bread characterization

Breads (B_C2.27_, B_C5.7_) made with sourdough (S_C5.7_, S_C2.27_) were compared with the control bread containing baker’s yeast (B_CTR_) (Table 5).

As expected, the pH values of B_C2.27_ and B_C5.7_ were significantly lower than in the control bread (B_CTR_) (*p* < 0.05). The TTA of B_C2.27_ was significantly higher than the other breads (*p* < 0.05), with a ca. 5-fold higher than B_CTR_. The lactic acid concentrations of breads containing sourdough were not significantly different (*p* > 0.05). The acetic acid was detectable only in B_C2.27_, where the QF was 3.61 ± 0.08.

The textural properties of breads were determined after baking. The use of sourdoughs affected the specific volume, which was ca. 10% lower in B_C2.27_ and B_C5.7_ compared to B_CTR_ (Table 5). No significant (*p* > 0.05) differences were found between B_C2.27_ and B_C5.7_.

The hardness lowest in B_CTR_. When S_C2.27_ was used, the value was significantly (*p* < 0.05) lower than B_C5.7_. Sourdough fermentation caused a significant (*p* < 0.05) decrease of the resilience and fracturability (Table 5). Moreover, the resilience and fracturability of B_C2.27_ were significantly (*p* < 0.05) higher than in B_C5.7_.

**Figure 3:**
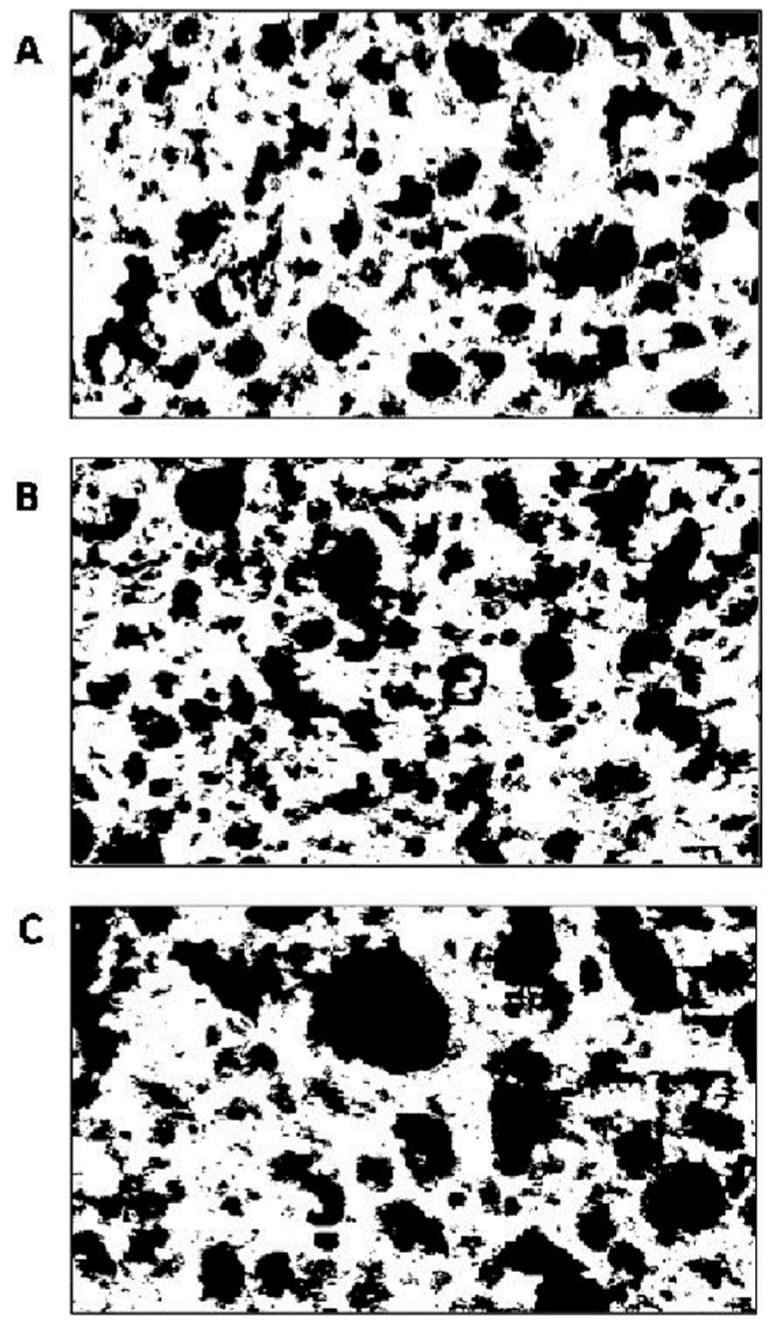
Representative digital images of experimental breads B_C2.27_ (**A**), B_C5.7_ (**B**) and B_CTR_ (**C**), showing the computed binary results from gray level thresholding at the two-cluster.

The crumb structure of breads was evaluated by image analysis technology (Figure 3, Table 5). The cell-total area (corresponding to the black pixel total area) of B_C2.27_ and B_C5.7_ was ca. 11% lower than the area of the control. The crust lightness (L) of B_C2.27_ and B_C5.7_ was lower than B_CTR_. Significant differences were observed for the colorimetric parameters, in particular, lightness (L) was significantly lower (*p* < 0.05) in sourdough breads compared to B_CTR_, while ΔE was up to 20% higher in the breads with sourdough compared to the control.

### 3.4. Effect of Salt Addition

**Figure 4:**
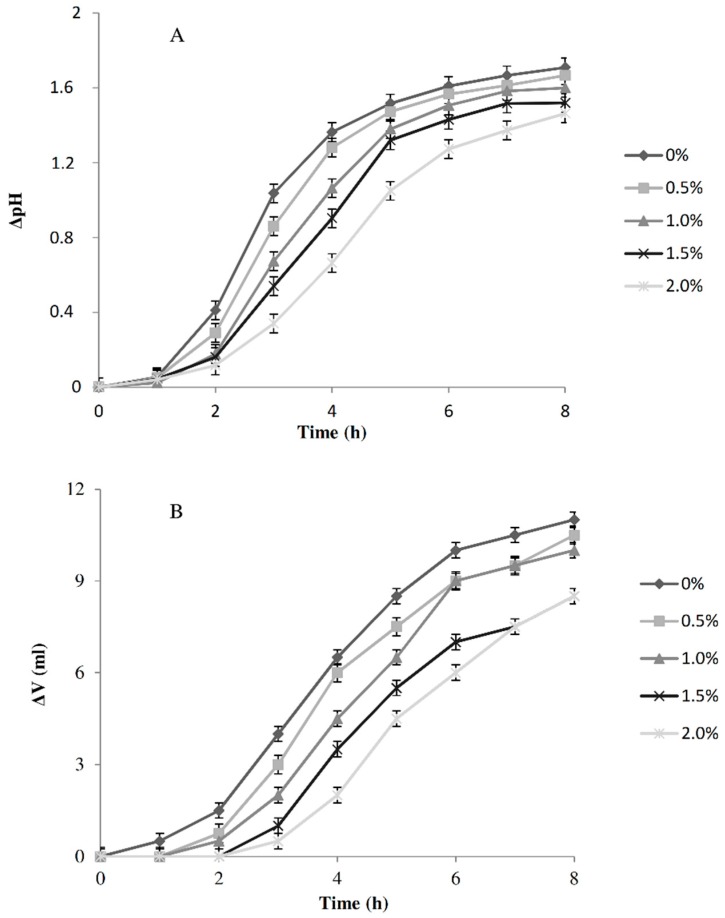
Variation of pH values (ΔpH, pH units) (**A**) and volume increase (ΔV, mL) (**B**) of doughs (DY 160) started with *L. citreum* C2.27 (initial cell number 7 log cfu/g), containing different percentages of salt: 0%, 0.5%, 1.0%, 1.5% and 2.0% (*w*/*w*). Fermentation was carried out at 30 °C for 8 h.

The effect of salt concentration on the acidification and leavening capacities of the strain *L. citreum* C2.27 was evaluated (Figure 4). The initial pH of the dough was 5.86 ± 0.02. As expected, the pH decreased over time, however, when the salt concentration was higher the ΔpH was lower. Salt concentration also affected the volume of the doughs. Additionally in this case, the higher volume increases were observed in doughs with lower salt concentrations. After 4 h of fermentation, the addition of 2% and 1.5% of salt caused ca. a 70 and 45% decrease of ΔV, respectively. On the basis of such results, in accordance with the bakery requirements regarding a short leaving time (4 h) and the need to not completely eliminate the salt, the bread-making at the pilot plant scale was carried out comparing the products containing 1.5% of salt with those without salt.

### 3.5. Bread-Making at the Industrial Pilot Scale

#### 3.5.1. Characterization of Sourdough and Puccia Doughs 

Based on the results obtained in laboratory, *L. citreum* C2.27 was selected and used as a starter in the bakery tests (Table 6). The initial pH value of the sourdough was ca. 6.20 and the fermentation determined its relevant significant decrease (*p* < 0.05).

The pH values of the bread doughs, including sourdough, were lower than those of the control doughs started with baker’s yeast after 4 h of fermentation. Moreover, the TTA and concentration of lactic and acetic acids were significantly higher in the inoculated doughs than in the control doughs. A low amount of lactic acid was detectable only in the control dough without salt. The concentration of lactic acid was significantly higher in the inoculated dough without salt addition (D0_C2.27_) than in D1.5_C2.27_, while the amounts of acetic acid and the QF did not significantly (*p* > 0.05) differ.

#### 3.5.2. Microbiological Analyses

Overall, the total LAB population in the sourdough was higher than 8 log cfu/g, and it was represented mainly by the starter LAB strain (Table 7). This value did not significantly (*p* > 0.05) differ from that which was found under laboratory conditions. Besides the starter, two strains belonging to *Lb. rossiae* and *Lb. plantarum* were found in the sourdough. The density of total LAB, as well as the density of the starter, were not significantly (*p* > 0.05) different in the puccia bread doughs containing or not containing salt (D1.5_C2.27_ and D0_C2.27_). The starter constituted ca. 81% of total LAB isolates analyzed. The *Lb. rossiae* strain isolated from sourdough was also isolated from both the bread doughs (D1.5_C2.27_ and D0_C2.27_).

The control bread doughs harbored a final LAB population at densities higher than 6 log cfu/g of LAB. Three strains belonging to the species *Lb. rossiae*, *L. mesenteroides* and *Lb. plantarum* were present in the dough D0_CTR_, while the dough containing salt (D1.5_CTR_) contained a different strain of *Lb. plantarum* and the *Lb. rossiae* strain which was also isolated from D0_C2.27_ and D1.5_C2.27_.

Yeasts, which were not found in the laboratory sourdough, were at 4 log cfu/g in the pilot plant sourdough. Molecular analysis allowed the differentiation of 3 strains belonging to the species *Wickerhamomyces anomalus*, *Candida pararugosa* and *C. parapsilosis*. *S. cerevisiae*, *C. pararugosa*, *Rhodotorula mucillaginosa*, *C. parapsilosis* (the same strain found in the sourdough) and *Hyphopichia burtonii* were the species identified in D0_C2.27_ and D1.5_C2.27_. *S. cerevisiae* constituted ca. 25% of the yeasts isolated from doughs. As expected, *S. cerevisiae* was the only yeast found at a very high density in control doughs. A single strain was identified in that population and it was the same found in the doughs made with sourdough. Moulds were not present in the sourdough and bread doughs.

#### 3.5.3. Puccia Bread Characterization

**Figure 5:**
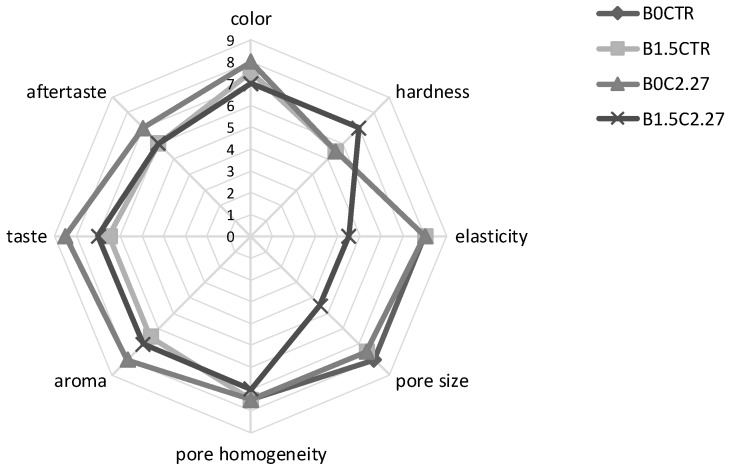
Spider web chart of the sensory analysis for puccia breads made in the bakery with liquid sourdough, without salt (B0_C2.27_) or with 1.5% salt (B1.5_C2.27_), and puccia breads made with baker’s yeast, without salt (B0_CTR_) or with 1.5% salt (B1.5_CTR_).

The bakery puccia breads produced with the selected sourdough, with (B1.5_C2.27_) or without (B0_C2.27_) salt, were compared with the control puccia breads (B1.5_CTR_ and B0_CTR_) leavened with baker’s yeast (Figure 5 and Table 6).

The pH values of B0_C2.27_ and B1.5_C2.27_ were slightly lower (*p* > 0.05) than those of the control puccia breads, while their TTA values were significantly higher than those of controls (*p* < 0.05).

The puccia bread without salt (B0_C2.27_) had higher concentrations of lactic and acetic acids than B1.5_C2.27_ (*p* < 0.05). The organic acids were not detectable (<LOD) in B0_CTR_ and B1.5_CTR_. The QF for B0_C2.27_ and B1.5_C2.27_ was below 3.5, but significantly higher in B1.5_C2.27_.

The puccia breads were subjected to sensory analysis (Figure 5). Not significant (*p* > 0.05) differences were observed in color intensity among the breads, although a slightly higher score was attributed to B0_C2.27_. Similar (*p* > 0.05) scores were observed for the perceived hardness and elasticity of B0_CTR_, B1.5_CTR_ and B0_C2.27_, while B1.5_C2.27_ was characterized by values significantly (*p* < 0.05) higher and lower for the two parameters, respectively. Compared to the other breads, B1.5_C2.27_ was also characterized by the lowest values for pore size (score of 4.5 compared to 8). Assessors attributed to aroma and taste scores ≥7 for all the breads, nevertheless, both the attributes were perceived with higher (*p* < 0.05) intensity in B0_C2.27_ compared to the other breads. The highest perception of aftertaste was also found in B0_C2.27_.

## 4. Discussion

Since LAB decisively influences the technological, nutritional, organoleptic and preservation properties of bakery products, their use has long been considered as an excellent strategy to improve the characteristics of those goods [32]. In this study, the possibility of exploiting LAB characteristics to obtain dough leavening in absence of baker’s yeast was investigated. For this purpose, LAB strains were characterized and selected, and a proper biotechnological protocol for the production of bakery products was developed and defined. This protocol was based on the use of a single starter strain to produce a ready-to-use liquid sourdough which would be directly added as an ingredient to the dough for bread production. Liquid sourdough responds to the industry demand for shorter, easier and more controllable procedures compared to traditional ones [17]. Moreover, the possibility of using a starter culture in the bakery product process is also desirable to obtain the control and the standardization of both the process and the product. 

### 4.1. Selection of the Starter Based on Technological and Microbiological Properties 

The strains characterized in this work were isolated from the cereal environment to increase the possibility of selecting strains which are more able to compete in the fermentation process [9], thus overcoming the difficulty often encountered in the use of a starter, concerning the possibility of optimal starter performance under the process conditions. The first selection was carried out on twelve strains and was mainly based on their acidification and leavening capabilities in the doughs. In this regard, it is interesting to note that the two most promising strains, *L. citreum* C2.27 and *W. confusa* C5.7, were both isolated from durum wheat semolina. Moreover, it is also noteworthy that the *Leuconostoc* and *Weissella* species are often isolated from sourdough ecosystems [12,16], and that these two heterofermentative strains have been previously characterized for their antifungal properties [19]. 

Although both selected strains were able to acidify and leaven the doughs, *L. citreum* C2.27 caused a greater increase in dough volume and an optimal fermentation quotient in the laboratory bread-making tests [33,34]. Moreover, *W. confusa* C5.7 showed greater difficulty and variability in dominating the other bacteria present in the doughs, and in particular it was associated with a strain of *Lb. plantarum*. On the contrary, *L. citreum* C2.27 gave positive and replicable results, as it was the only strain identified in both the sourdough and the dough. This strain was able to fully dominate the lactic population naturally present in the flour, also completely avoiding the development of endogenous yeasts.

No differences were found in the specific volume of breads obtained using the strains C2.27 or C5.7 (for both, it was slightly lower than that of the control bread). Nevertheless, C2.27 obtained better textural properties than those obtained using the strain C5.7. Indeed, according to the instrumental analysis of the technological features, the bread produced with *L. citreum* C2.27 was more soft and elastic than that produced with *W. confusa* C5.7. Therefore, *L. citreum* C2.27 was considered more suitable than *W. confusa* C5.7 to make a yeast-free bread, and it was applied in an industrial bakery for the production of the puccia bread without baker’s yeast. 

### 4.2. Bread-Making at Industrial Pilot Scale 

The biotechnological protocol was adapted to the needs of the bakery related to the leavening times and the formulation for the production of the typical Apulian bread, “puccia salentina”. This is a small, round and flat bread, characterized by an intermediate specific volume, which is typically used as a stuffed bread with different ingredients. The trials confirmed the applicability of the biotechnological protocol and the suitability of the selected strain *L. citreum* C2.27 in order to produce a puccia bread without baker’s yeast. In fact, the total lactic population of sourdough was above 10^8^ cfu/g, typical for sourdoughs [16], and the starter strain was dominant, even if it was not the only one present. Unlike the results obtained in the laboratory tests, the yeasts were present in the sourdough, but the overall amount was very low in comparison with the density normally found in that environment (up to 10^7^ cfu/g) [16], and *S. cerevisiae* was not present. Certainly, the use of a liquid ready-to-use sourdough avoided the increase in yeasts observed by Corona et al. [12] during the refreshments for the sourdough production. A minimal presence of *S. cerevisiae* was detected in the bakery bread doughs prepared with the liquid sourdough, attributable to environmental contaminations which could be eliminated by using a separated production line for yeast-free products. As highlighted by several authors, the presence of *S. cerevisiae* could be attributed to the daily use of baker’s yeast in bakeries [35,36]. As matter of fact, all the isolates of *S. cerevisiae* analyzed in this study had the same rep-PCR electrophoretic profile of the strain present in the doughs prepared with commercial baker’s yeast. 

It is important to note that the sensory analyses did not reveal particular differences for the structural parameters between the puccia bread obtained with baker’s yeast and that obtained with sourdough, especially when salt was not used. Indeed, it is known that salt affects the dough ecosystem [37] and lactic acid bacteria growth [38], therefore, as expected, the acidification and leavening capabilities of *L. citreum* C2.27 were negatively affected by salt. In this case, despite the lack of salt, the bread’s taste and aroma were very intense. The high concentrations of total free amino acids, and in particular of aspartic and glutamic acids, mainly responsible for such an effect. The concentration of free amino acids and their derivatives (including volatiles) strongly depends from the proteolytic activity of the LAB [25]. The key role of LAB fermentation in defining the more complex flavor of the sourdough compared to that obtained only with baker’s yeast is well known [25,39], and in particular, the use of heterofermentative LAB was previously associated with a good aromatic profile [11]. The high concentration of free amino acids, available for the Maillard reaction during baking, also explain the more intense color of the sourdough breads observed through instrumental and sensory analyses [14]. Therefore, the successful combination of the proposed biotechnological protocol for salt removal is also in agreement with a current trend oriented towards the reduction of salt in food.

## 5. Conclusions

In conclusion, the selected strain *L. citreum* C2.27 (and its application according to the procedure developed and defined in this study) allowed for the production of bread without baker’s yeast, with good sensory characteristics and with an appearance similar to conventional bread, thus intercepting the current demand from bakers and consumers and reducing the occurrence of possible food adverse reactions.

## Figures and Tables

**Table 1:** Strains used and their corresponding characteristics.

Species	Strain	Type of Fermentation	Culture Collection	Isolation Source	Culture Medium
*Lb. plantarum*	21B [18]	FHe	ISPA-CNR	Sourdough	MRS
*Lc. lactis*	C5.6 [19]	OHo	ISPA-CNR	Durum wheat semolina	mMRS [18]
*W. confusa*	C5.7 [19]	OHe	ISPA-CNR	Durum wheat semolina	MRS
*L. citreum*	C2.27 [19]	OHe	ISPA-CNR	Durum wheat semolina	MRS
*W. cibaria*	C21.4 [19]	OHe	ISPA-CNR	Durum wheat semolina	mMRS
*Lb. brevis*	18F	OHe	ISPA-CNR	Sourdough	MRS
*P. pentosaceus*	LM	FHe	ISPA-CNR	Durum wheat semolina	MRS
*Lb. plantarum*	DC400	FHe	DiSSPA	Sourdough	MRS
*Lc. lactis*	10γ	OHo	DiSSPA	Sourdough	mMRS
*W. cibaria*	5S	OHe	DiSSPA	Sourdough	mMRS
*Lb. rossiae*	DSM15814	OHe	DSMZ	Sourdough	mMRS
*W. confusa*	DSM20196	OHe	DSMZ	Sugar cane	MRS

*Lb.*, *Lactobacillus*; *L.*, *Leuconostoc*; *Lc.*, *Lactococcus*; *W.*, *Weissella*; *P.*, *Pediococcus*. FHe, facultative heterofermentative; OHe, obligate heterofermentative; Oho, obligate homofermentative; MRS, de Man–Rogosa–Sharpe broth; mMRS, modified MRS. ISPA-CNR, Institute of Sciences of Food Production of National Research Council, Bari, Italy; DiSSPA, Department of Soil, Plant and Food Science, University of Bari, Italy; DSMZ, DSMZ-German Collection of Microorganisms and Cell Cultures, Leibniz Institute, Braunschweig, Germany.

**Table 2:** Acidification (ΔpH, pH units), volume increase (ΔV, mL) and total titratable acidity (TTA, mL di NaOH 0.1 N/10 g) of doughs singly inoculated with the lactic acid bacteria (LAB) strains, as determined after 8, 16 and 24 h of incubation at 30 °C.

Species	Strain	ΔpH *	ΔV	TTA	ΔpH	ΔV	TTA	ΔpH	ΔV	TTA
		8 h			16 h			24 h		
*Lb. plantarum*	21B	2.10 ± 0.01 ^e^	0 ^a^	4.33 ± 0.17 ^cde^	2.32 ± 0.01 ^d^	0 ^a^	7.87 ± 0.13 ^ef^	2.44 ± 0.04 ^de^	0 ^a^	9 ± 0.3 ^fg^
*Lc. lactis*	C5.6	1.72 ± 0.01 ^d^	0 ^a^	3.50 ± 0.29 ^abc^	1.84 ± 0.00 ^a^	0 ^a^	3.33 ± 0.33 ^a^	1.97 ± 0.01 ^a^	0 ^a^	4.67 ± 0.33 ^a^
*W. confusa*	C5.7	1.68 ± 0.02 ^cd^	7.50 ± 0.00 ^d^	4.17 ± 0.17 ^bcde^	1.95 ± 0.03 ^ab^	8.75 ± 0.72 ^b^	6.00 ± 0.00 ^bc^	2.02 ± 0.02 ^a^	10.75 ± 1.01 ^b^	5.00 ± 0.58 ^ab^
*L. citreum*	C2.27	2.06 ± 0.01 ^e^	9.83 ± 0.17 ^e^	4.67 ± 0.33 ^de^	2.28 ± 0.02 ^d^	13.90 ± 2.31 ^cd^	9.00 ± 0.00 ^f^	2.37 ± 0.03 ^cde^	17.17 ± 1.67 ^c^	9.67 ± 0.17 ^g^
*W. cibaria*	C21.4	1.73 ± 0.10 ^d^	6.50 ± 0.00 ^d^	4.83 ± 0.44 ^de^	2.10 ± 0.05 ^bc^	8.00 ± 0.58 ^b^	6.50 ± 0.29 ^bcd^	2.05 ± 0.02 ^a^	7.75 ± 0.14 ^b^	6.33 ± 0.33 ^bcd^
*Lb. brevis*	18F	0.80 ± 0.07 ^a^	1.33 ± 0.17 ^b^	3.77 ± 0.27 ^abcd^	1.84 ± 0.08 ^a^	11.10 ± 0.74 ^bc^	5.63 ± 0.20 ^bc^	2.24 ± 0.02 ^b^	17.33 ± 0.88 ^c^	7.27 ± 0.14 ^de^
*P. pentosaceus*	LM	1.76 ± 0.00 ^d^	0 ^a^	2.83 ± 0.17 ^a^	2.26 ± 0.01 ^d^	0 ^a^	7.33 ± 0.33 ^de^	2.34 ± 0.01 ^bcd^	0 ^a^	7.17 ± 0.17 ^de^
*Lb. plantarum*	DC400	1.63 ± 0.02 ^cd^	0 ^a^	3.00 ± 0.00 ^a^	2.34 ± 0.01 ^d^	0 ^a^	5.33 ± 0.33 ^b^	2.40 ± 0.01 ^cde^	0 ^a^	8.00 ± 0.58 ^ef^
*Lc. lactis*	10γ	1.68 ± 0.01 ^cd^	0 ^a^	3.33 ± 0.17 ^abc^	2.22 ± 0.01 ^cd^	0 ^a^	6.50 ± 0.29 ^bcd^	2.31 ± 0.01 ^bc^	0 ^a^	6.83 ± 0.16 ^cde^
*W. cibaria*	5S	1.29 ± 0.05 ^b^	2.50 ± 0.00 ^c^	3.17 ± 0.17 ^ab^	2.33 ± 0.01 ^d^	17.50 ± 0.00 ^de^	8.17 ± 0.17 ^ef^	2.45 ± 0.03 ^e^	19.25 ± 0.14 ^c^	9.00 ± 0.00 ^fg^
*Lb. rossiae*	DSM 15814	1.48 ± 0.10 ^bc^	2.50 ± 0.00 ^c^	3.00 ± 0.00 ^a^	2.28 ± 0.01 ^d^	18.25 ± 0.72 ^e^	6.67 ± 0.17 ^cd^	2.47 ± 0.02 ^e^	20.00 ± 0.00 ^c^	9.00 ± 0.00 ^fg^
*W. confusa*	DSM 20196	1.61 ± 0.01 ^cd^	8.75 ± 0.72 ^e^	5.17 ± 0.17 ^e^	2.09 ± 0.02 ^bc^	11.00 ± 0.29 ^bc^	5.83 ± 0.17 ^bc^	2.07 ± 0.02 ^a^	10.00 ± 0.00 ^b^	5.33 ± 0.33 ^abc^

Data represent means of three independent experiments ± standard error. ^a–g^ Values in the same column with different letters differ significantly (*p* < 0.05). * The initial pH of the doughs was 6.04 ± 0.22. *Lb.*, *Lactobacillus*; *L.*, *Leuconostoc*; *Lc.*, *Lactococcus*; *W.*, *Weissella*; *P.*, *Pediococcus*.

**Table 3:** Chemicophysical characteristics of liquid sourdoughs (S_C2.27_ and S_C5.7_) inoculated with starter strains (*L. citreum* C2.27 or *W. confusa* C5.7) and bread doughs started with the liquid sourdoughs (D_C2.27_ and D_C5.7_) or with baker’s yeast (D_CTR_) in laboratory tests.

Characteristics	Liquid Sourdough		Bread Dough		
	S_C2.27_	S_C5.7_	D_C2.27_	D_C5.7_	D_CTR_
pH	3.48 ± 0.05 ^a^	3.56 ± 0.05 ^a^	4.29 ± 0.05 ^b^	4.30 ± 0.04 ^b^	5.18 ± 0.22 ^a^
ΔpH	2.69 ± 0.07 ^a^	2.60 ± 0.09 ^a^	0.89 ± 0.05 ^a^	0.92 ± 0.07 ^a^	0.30 ± 0.07 ^b^
ΔV (mL)	-	-	8.10 ± 0.24 ^b^	4.80 ± 0.59 ^b^	20.42 ± 2.45 ^a^
TTA (mL)	4.10 ± 0.10 ^a^	3.60 ± 0.10 ^a^	5.80 ± 0.43 ^a^	4.70 ± 0.44 ^a^	1.75 ± 0.02 ^b^
Lactic acid (mmol/kg)	6.53 ± 0.16 ^a^	4.27 ± 0.34 ^b^	13.68 ± 0.42 ^a^	10.79 ± 0.24 ^b^	ND
Acetic acid (mmol/kg)	2.17 ± 0.04 ^a^	0.92 ± 0.07 ^b^	6.48 ± 0.24	ND	ND
QF	3.01 ± 0.10 ^a^	4.77 ± 0.77 ^a^	2.11 ± 0.11	-	-
Peptides (g/kg)	7.36 ± 0.31 ^b^	7.92 ± 0.11 ^a^	9.46 ± 0.11 ^a^	9.76 ± 0.68 ^a^	4.25 ± 0.54 ^b^
Total Free Amino Acids (mg/kg)	693 ± 16 ^b^	894 ± 17 ^a^	753 ± 7 ^b^	856 ± 8 ^a^	428 ± 6 ^c^

Data represent means of three independent experiments ± standard error. ^a–c^ Values refer to liquid sourdough or to bread dough in the same row, where different letters differ significantly (*p* < 0.05). ND: Not detected (<LOD, limit of detection), QF: Quotient of fermentation.

**Table 4:** Microbiological characteristics of liquid sourdoughs (S_C2.27_ and S_C5.7_) inoculated with selected starters (*L. citreum* C2.27 or *W. confusa* C5.7) and bread doughs obtained with the liquid sourdoughs (D_C2.27_ and D_C5.7_) or with baker’s yeast (D_CTR_) in laboratory tests. The microbial loads (log cfu/g) of lactic acid bacteria (LAB) and of the starter strains, yeasts and moulds, as well as species of bacteria and yeasts present in sourdoughs and bread doughs are reported.

Sample	LAB (log cfu/g)	Starter Strain (log cfu/g)	Other LAB Species §	Yeasts (log cfu/g)	Moulds (log cfu/g)	Yeast Species
**S_C2.27_**	8.94 ± 0.28 ^a^	8.94 ± 0.28 ^a^	-	-	0.60 ± 0.09 ^a^	-
**S_C5.7_**	8.43 ± 0.24 ^a^	8.27 ± 0.28 ^a^	*Lb. plantarum* (1) *	-	0.90 ± 0.21 ^a^	-
**D_C2.27_**	8.83 ± 0.17 ^a^	8.83 ± 0.17 ^a^	-	-	-	-
**D_C5.7_**	8.63 ± 0.17 ^a^	6.88 ± 1.83 ^a^	*Lb. plantarum* (1) *	1.80 ± 0.40 ^b^	1.79 ± 0.79	*F. magnum*/*F. floriforme* (1)
**D_CTR_**	5.57 ± 0.23 ^b^	-	*Lb. sakei* (1)	7.99 ± 0.23 ^a^	-	*S. cerevisiae* (1)
			*Lb. curvatus* (2)			
			*L. gelidum* (1)			
			*L. pseudomesenteroides* (1)			
			*Lb. brevis* (1)			
			*Lb. paraplantarum* (2)			
			*Lb. paracasei* (1)			

Data represent means of three independent experiments ± standard error. ^a,b^ Values refer to liquid sourdough or to bread dough in the same column, where different letters differ significantly (*p* < 0.05). *Lb.*, *Lactobacillus*; *L.*, *Leuconostoc*; *S.*, *Saccharomyces*; *F.*, *Filobasidium*. **§** Bacterial species identified in liquid sourdoughs and bread doughs that were different from those of the starter strains (*L. citreum* and *W. confusa*). * The same single strain was identified in S_C5.7_ and in D_C5.7_. (1) A single strain was identified on the basis of the rep-PCR profile. (2) Two different strains belonging to the same species were identified on the basis of the rep-PCR profiles.

**Table 5:** Characteristics of breads made with liquid sourdough (B_C2.27_ and B_C5.7_) or with baker’s yeast (B_CTR_) in laboratory tests.

Characteristics of Breads	Bread		
	B_C2.27_	B_C5.7_	B_CTR_
pH	4.48 ± 0.04 ^b^	4.61 ± 0.001 ^b^	5.52 ± 0.15 ^a^
TTA (mL)	5.80 ± 0.42 ^a^	3.06 ± 0.15 ^b^	1.15 ± 0.01 ^c^
Lactic Acid (mmol/kg)	15.52 ± 0.25 ^a^	16.30 ± 0.81 ^a^	ND
Acetic Acid (mmol/kg)	4.30 ± 0.01	ND	ND
QF	3.61 ± 0.08	-	-
a_w_	0.979 ± 0.002 ^a^	0.975 ± 0.002 ^a^	0.977 ± 0.000 ^a^
Specific volume (cm^3^/g)	2.21 ± 0.1 ^b^	2.20 ± 0.2 ^b^	2.46 ± 0.2 ^a^
*Textural profile analysis*			
Hardness (g)	7050 ± 63 ^b^	7564 ± 66 ^a^	6034 ± 52 ^c^
Resilience	0.67 ± 0.04 ^b^	0.61 ± 0.04 ^c^	0.76 ± 0.05 ^a^
Fracturability	5579 ± 36 ^b^	5210 ± 49 ^c^	5745 ± 51 ^a^
*Image analysis*			
Black pixel area * (%)	34.8 ± 0.3 ^b^	34.9 ± 0.1 ^b^	38.9 ± 0.1 ^a^
*Color analysis*			
L	51.9 ± 1.3 ^b^	47.4 ± 8.8 ^c^	60.1 ± 2.2 ^a^
a	9.9 ± 1.2 ^a^	8.3 ± 1.4 ^a^	5.5 ± 1.3 ^b^
b	32.6 ± 1.6 ^a^	31.3 ± 0.8 ^a^	31.1 ± 0.5 ^a^
ΔE	52.3 ± 1.2 ^b^	54.7 ± 1.9 ^a^	44.1 ± 1.3 ^c^

Data represent means of three independent experiments ± standard error. ^a–c^ Values in the same row with different letters differ significantly (*p* < 0.05). * The gas cell area is expressed as the percentage of black pixel to total area of the image. ND: Not detected (<LOD).

**Table 6:** Chemical characteristics of liquid sourdough (S_C2.27_) and puccia bread doughs made with liquid sourdough, without salt (D0_C2.27_) or with 1.5% salt (D1.5_C2.27_), bread doughs made with baker’s yeast, without salt (D0_CTR_) or with 1.5% salt (D1.5_CTR_), and puccia breads (B0_C2.27_, B1.5_C2.27_, B0_CTR_, B1.5_CTR_) obtained by baking of the bread doughs in bakery tests.

Chemical Characteristics	Sourdough	Bread Dough				Puccia Bread			
	S_C2.27_	D0_C2.27_	D1.5_C2.27_	D0_CTR_	D1.5_CTR_	B0_C2.27_	B1.5_C2.27_	B0_CTR_	B1.5_CTR_
pH	3.57 ± 0.05	4.58 ± 0.15 ^a^	4.78 ± 0.28 ^ac^	5.48 ± 0.05 ^b^	5.44 ± 0.06 ^bc^	4.98 ± 0.08 ^a^	5.53 ± 0.07 ^a^	5.94 ± 0.34 ^a^	5.72 ± 0.02 ^a^
TTA (mL)	5.53 ± 0.24	6.55 ± 0.81 ^a^	5.55 ± 0.04 ^a^	2.90 ± 0.03 ^b^	3.03 ± 0.04 ^b^	4.60 ± 0.81 ^a^	3.05 ± 0.32 ^a^	1.85 ± 0.37 ^b^	1.80 ± 0.30 ^b^
Lactic acid (mmol/Kg)	15.82 ± 0.02	23.04 ± 0.25 ^a^	19.02 ± 0.39 ^b^	2.31 ± 0.18 ^c^	ND	14.29 ± 0.91 ^a^	8.42 ± 0.45 ^b^	ND	ND
Acetic acid (mmol/Kg)	8.23 ± 0.15	7.05 ± 0.95 ^a^	5.73 ± 0.12 ^a^	ND	ND	5.67 ± 0.63 ^a^	2.52 ± 0.05 ^b^	ND	ND
QF	1.92 ± 0.03	3.31 ± 0.02 ^a^	3.39 ± 0.46 ^a^	-	-	2.60 ± 0.04 ^b^	3.34 ± 0.87 ^a^	-	-

Data represent means of three independent experiments ± standard error. ^a–c^ Values refer to bread doughs or to breads in the same row, where different letters differ significantly (*p* < 0.05). ND: Not detected (<LOD).

**Table 7:** Microbiological characteristics of liquid sourdough (S_C2.27_) inoculated with the starter strain and puccia bread doughs made with liquid sourdough, without salt (D0_C2.27_) or with 1.5% salt (D1.5_C2.27_), and puccia bread doughs made with baker’s yeast, without salt (D0_CTR_) or with 1.5% salt (D1.5_CTR_), in bakery tests. The microbial loads (log cfu/g) of lactic acid bacteria (LAB) and of starter strain *L. citreum* C2.27, yeasts and moulds, as well as species of bacteria and yeasts present in sourdough and puccia bread doughs are reported.

Sample	LAB (log cfu/g)	Starter Strain (log cfu/g)	Other LAB Species §	Yeasts (log cfu/g)	Moulds (log cfu/g)	Yeast Species
**S_C2.27_**	8.90 ± 0.04	8.72 ± 0.02	*Lb. rossiae* (1) *	4.22 ± 0.06	-	*W. anomalus* (1) *C. pararugosa* (1) *C. parapsilosis* (1) **
			*Lb. plantarum* (1)			
**D0_C2.27_**	8.91 ± 0.04 ^a^	8.76 ± 0.08 ^a^	*Lb. rossiae* (1) *	3.82 ± 0.01 ^b^	-	*S. cerevisiae* (1) *** C. pararugosa (1) *R. mucillaginosa* (1) *C. parapsilosis* (1) ** H. burtonii (1)
**D1.5_C2.27_**	8.91 ± 0.09 ^a^	8.85 ± 0.07 ^a^		3.95 ± 0.06 ^b^	-	
**D0_CTR_**	6.68 ± 0.02 ^b^	-	*L. mesenteroides* (1) *Lb. plantarum* (2) *Lb. rossiae* (1) *	7.97 ± 0.01 ^a^		*S. cerevisiae* (1) ***
**D1.5_CTR_**	6.82 ± 0.18 ^b^	-		8.03 ± 0.14 ^a^		

Data represent the means of three independent experiments ± standard error. ^a,b^ Values refer to bread doughs in the same column, where different letters differ significantly (*p* < 0.05); *Lb.*, *Lactobacillus*; *L.*, *Leuconostoc*; *C.*, *Candida*; *S.*, *Saccharomyces*; *R.*, *Rhodotorula*; *H.*, *Hyphopichia*; *W.*, *Wickerhamomyces*. **§** Bacterial speciesidentified in liquid sourdough and bread doughs (D0 _C2.27_/D1.5_C2.27_ and D0_CTR_/D1.5_CTR_) different from that of the starter strain (*L. citreum*). * The same single strain was identified in S_C2.27_ and all the bread doughs; ** the same single strain was identified in S_C2.27_, D0_C2.27_ and D1.5_C2.27_; *** the same single strain was identified in all the doughs. (1) Based on the rep-PCR profile, a single strain was identified. (2) Based on the rep-PCR profiles, two different strains belonging to the same species were identified.
